# Directional microwave ablation in spine: experimental assessment of computational modeling

**DOI:** 10.1080/02656736.2024.2313492

**Published:** 2024-02-18

**Authors:** Austin Pfannenstiel, Haileigh Avellar, Clay Hallman, Brandon L. Plattner, Margaret A. Highland, Francois H. Cornelis, Warren L. Beard, Punit Prakash

**Affiliations:** a Precision Microwave Inc, Manhattan, KS, USA; b Department of Electrical and Computer Engineering, KS State University, Manhattan, KS, USA; c Department of Clinical Sciences, Kansas State University, Manhattan, KS, USA; d Department of Diagnostic Medicine/Pathobiology and Kansas State Veterinary Diagnostic Laboratory, Kansas State University, Manhattan, KS, USA; e WI Veterinary Diagnostic Laboratory, University of Wisconsin-Madison, Madison, WI, USA; f Interventional Radiology Service, Memorial Sloan Kettering Cancer Center, NY, NY, USA

**Keywords:** Microwave ablation, bone ablation, bone tumor, thermal ablation, directional antenna

## Abstract

**Background:**

Despite the theoretical advantages of treating metastatic bone disease with microwave ablation (MWA), there are few reports characterizing microwave absorption and bioheat transfer in bone. This report describes a computational modeling-based approach to simulate directional microwave ablation (dMWA) in spine, supported by *ex vivo* and pilot *in vivo* experiments in porcine vertebral bodies.

**Materials and methods:**

A 3D computational model of microwave ablation within porcine vertebral bodies was developed. *Ex vivo* porcine vertebra experiments using a dMWA applicator measured temperatures approximately 10.1 mm radially from the applicator in the direction of MW radiation (T1) and approximately 2.4 mm in the contra-lateral direction (T2). Histologic assessment of ablated *ex vivo* tissue was conducted and experimental results compared to simulations. Pilot *in vivo* experiments in porcine vertebral bodies assessed ablation zones histologically and with CT and MRI.

**Results:**

Experimental T1 and T2 temperatures were within 3–7% and 11–33% of simulated temperature values. Visible ablation zones, as indicated by grayed tissue, were smaller than those typical in other soft tissues. Posthumous MRI images of *in vivo* ablations showed hyperintensity. *In vivo* experiments illustrated the technical feasibility of creating directional microwave ablation zones in porcine vertebral body.

**Conclusion:**

Computational models and experimental studies illustrate the feasibility of controlled dMWA in bone tissue.

## I. Introduction

Bone is the third most common site of metastatic spread [1] with bone involvement in 60–84% of patients with metastatic disease. Approximately 70% of those patients have resulting bone pain [1]. Bone metastases can also increase a patient’s risk of fractures, spinal cord compression, hypercalcemia, and other associated complications which may increase mortality [1].

Common treatments for metastatic disease include surgery, chemotherapy, and radiation therapy, with the goal to provide pain palliation and local control. Recently, the National Comprehensive Cancer Network Guidelines for Adult Cancer Pain added thermal ablation as a consideration for patients with metastatic bone pain in the absence of an oncologic emergency [2]. Local thermal ablation techniques have largely been used for palliative treatment of metastatic disease, where targeted tumor sizes can range widely from as small as 1 cm up to 10 cm in maximum linear dimension [3,4]. When employed for achieving local control, more favorable outcomes are achieved for tumors <2 cm in maximum dimension [5]. Both cryoablation (CA) and radiofrequency ablation (RFA) have been shown to be safe and highly effective in alleviating pain arising from metastases of the spine or other bones in several single-arm, prospective, multicenter trials [2,6]. Fewer clinical reports have reported the use of microwave ablation (MWA), despite its theoretical advantages of rapid treatment times and the ability to radiate through high impedance tissues, such as bone. Only a single, prospective, pilot study and some retrospective cases have shown MWA to provide safe and effective palliation and tumor necrosis [2]. Furthermore, there are few reports on the biophysics of microwave absorption and heat transfer in bone tissue [7,8].

MWA devices have largely been designed and optimized for creating large volume ablation zones in highly vascular tissue such as the liver [9]. However, bone tissue has substantially different electrical and thermal properties than liver and other soft tissues [10], and many of the prospective ablation sites are in close proximity to critical structures such as the spinal cord or nerve roots. Therefore, high-powered MWA applicators designed for large volume ablation in highly perfused organs may result in increased risk of inadvertent injury to adjacent critical healthy tissues when used in vertebrae. Recently, directional microwave ablation (dMWA) has been suggested to address these limitations by allowing to better spatially control the delivery of energy [9]. Building on preliminary results reported in Pfannenstiel et al. [11], the present report describes a computational modeling-based approach to simulate a dMWA applicator performance in the spine, supported by *ex vivo* and pilot *in vivo* experiments, to analyze the technical feasibility of using dMWA applicators for treating bone metastasis.

## II. Methods

### II.1 Applicator

The dMWA applicator design used for this study is an experimental 2.45 GHz, water-cooled, 14-gauge design which has been previously characterized in *ex vivo* and *in vivo* liver tissues [12]. The specific applicators used for the *in vivo* pilot were industrialized prototypes consisting of the same basic internal antenna design as the *ex vivo* applicators with additional usability and reliability-focused improvements to support their use in a clinically relevant setting. Improvements included a trocar tip, a rigid fiberglass shaft, an integrated MW transmission and water line bundle, and a custom generator connector.

### II.2 Computational models of MWA in vertebral body

A 3D finite element method (FEM) solver (COMSOL Multiphysics v5.5, COMSOL, INC. Burlington, MA) operating on a machine with an Intel Xeon W-2225 Processor operating at 4.1 GHz and 64 GB of RAM was used to model the electromagnetic radiation pattern and subsequent heat transfer from the MWA applicator in a manner similar to that reported by McWilliams et al. [13] and Sebek et al. [14].

**Figure 1.**
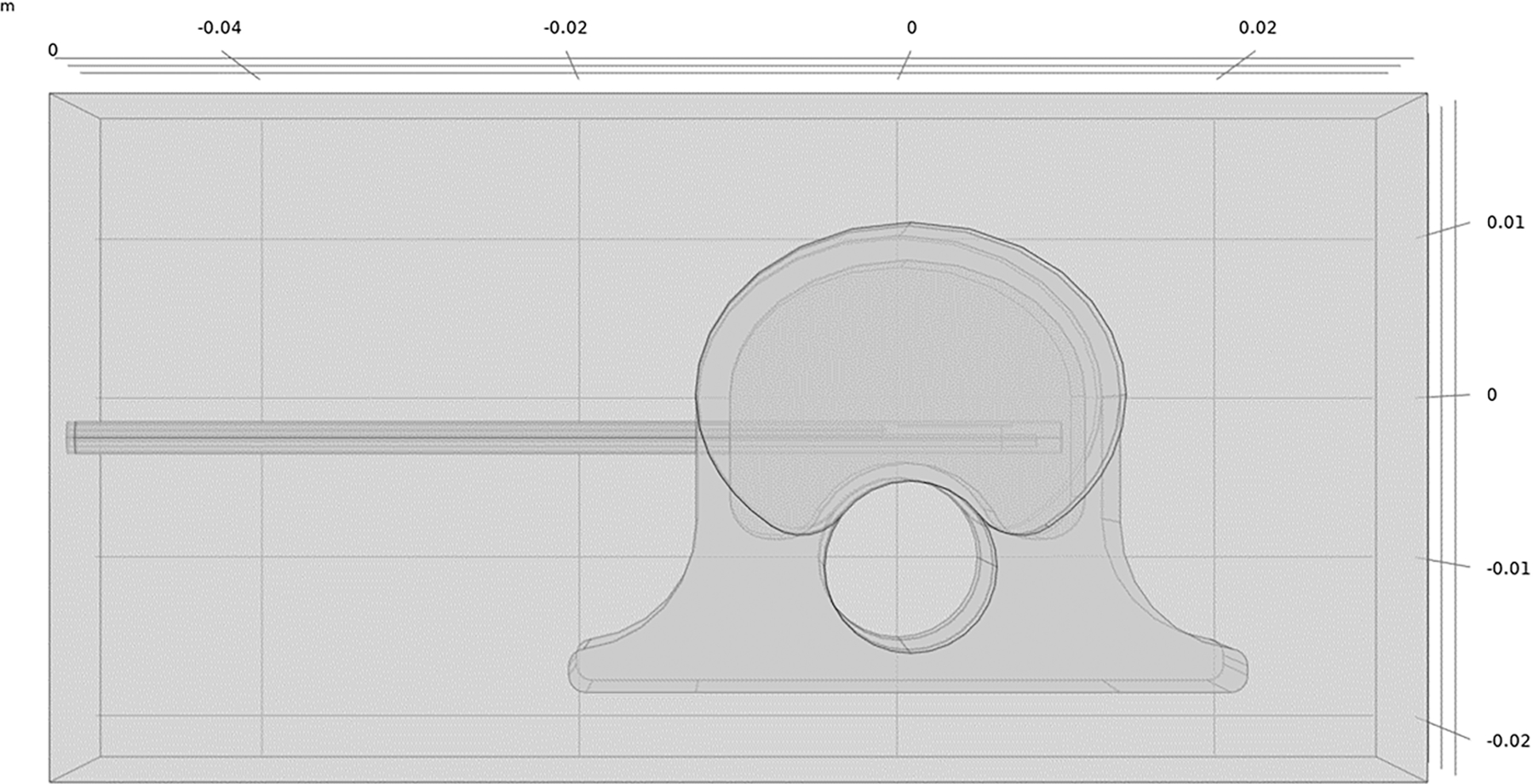
Model geometry used to simulate ablation with a dMWA device in the vertebral body of a 45 kg pig.

*Ex vivo* simulations used a vertebral body model developed in COMSOL to approximate a 45 kg pig as shown in Figure 1. Vertebral body dimensions were 2.5 cm wide (latero-lateral), 2.0 cm deep (dorsoventral), and 2.5 cm tall (craniocaudal); cortical bone surrounding cancellous bone was 2 mm thick; the spinal canal was 1.0 cm in diameter with spinal cord tissue properties; and the intervertebral disks, placed cranial and caudal to the vertebral body, were each 7.5 mm thick. The distal 6 cm of the dMWA applicator was modeled aimed in a ventral direction with an entry though the lateral wall of the vertebral body and the back of the applicator positioned approximately 2 mm from the spinal canal. The encompassing 8 cm wide × 4 cm tall × 4 cm deep domain was modeled as air to emulate our *ex vivo* experimental setup where soft tissue surrounding the vertebrae was removed. Although not a feasible clinical approach, the applicator insertion path shown in Figure 1 was selected to facilitate *ex vivo* experimentation in porcine tissue with relatively small vertebral bodies and without the aid of image guidance.

The material electromagnetic and thermal properties used in simulation are given in Table 1. The model assumed approximately 50% attenuation losses in interconnecting cabling and within the applicator based on measured and estimated values [–1.54 dB from 2.5 m of Succoform 141 (measured) + −0.17 dB from QMA to SMA adapter (datasheet) + −0.73 dB from 0.25 m of UT-034 (extrapolated from datasheet) + −0.20 dB from applicator SMA connector (estimated) + −0.20 from generator connector = −2.84 dB]. Simulations investigated applied powers of 40 and 60 W (80 and 120 W generator setpoint) for 3.5 or 5 min.

The initial temperature of the tissue domain was set to 20 °C or 37 °C for *ex vivo* simulations to model specimens at room temperature or heated back to physiologic temperatures in a water bath, respectively. Follow-on simulations were also conducted at an initial temperature of 30 °C, as it was observed that the 37 °C warmed *ex vivo* experimental specimens had cooled to an average temperature of 30.2 °C due to ambient loss during the time it took to remove them from the water bath, place the ablation applicator, and commence the ablation experiments. A convective heat transfer boundary condition with a heat transfer coefficient of 10 W/ (m^2^ K) and external temperature of 20 °C was modeled at the extents of the vertebrae to account for ambient losses. To simulate the forced cooling of the applicator, a 30 °C isothermal boundary condition was applied to the outer surface of the applicator. The use of isothermal boundary conditions to model forced cooling of the applicator has been previously proposed in literature [16] and is an approximation to account for microwave heating of the chilled cooling water near the antenna, heat transfer from attenuation losses in the applicator transmission cable, and heat transfer from the surrounding tissue to the applicator surface. Since the temperature dependence of the electrical and thermal variables of bone tissue have not been reported in the literature, simulations modeled tissue properties as static values. A non-uniform mesh with 695,630 tetrahedral elements was employed; this mesh was selected after iterative refinement to assure convergence of the output variables (i.e. electric field, temperature). The mesh density was highest around the input port of applicator, with a maximal tetrahedral element edge length of 0.01 mm. Element edge lengths were allowed to grow up to 0.2 mm within the applicator shaft. The mesh was coarsest in the regions furthest away from antenna and tissue boundaries with a maximum element edge length of 3 mm. An implicit transient solver was used with a maximum time step of 10 s, where at each step temperature dependent thermal and electrical properties were updated. The processing time was 50 min.

### II.3 Ex vivo MWA experiments in porcine vertebral body

**Figure 2.**
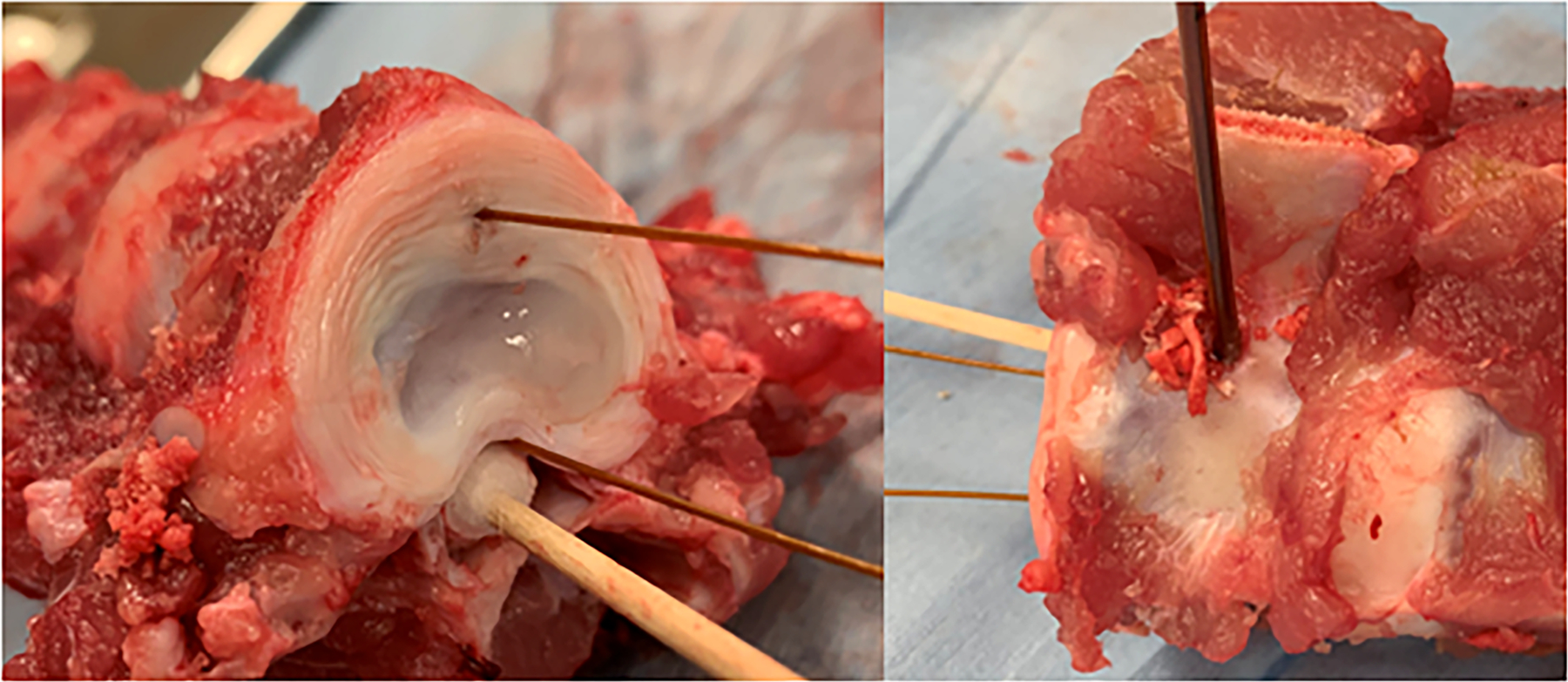
*Ex vivo* porcine model. Placement of temperature sensors (left) and dMWA applicator (right) within vertebral body.

Spinal columns were excised from approximately 45 kg pigs within 2 h from the time they were euthanized. The thoracic and lumbar sections of the spinal column were separated into segments of three vertebra. The ribs, transverse processes, and soft tissues surrounding the ventral and lateral sides of the vertebral bodies were removed. One set of vertebrae was allowed to cool to room temperature and a second was placed in plastic bags and warmed to 37 °C in a water bath. A cordless power drill with a 3/32” drill bit was used to provide a pathway for the dMWA applicator. The pathway was centered along, and perpendicular to, the craniocaudal axis on the lateral wall of the vertebral body, perpendicular and approximately 2–3 mm ventral to the spinal canal. The pathway extended through almost the entire width of the vertebral body, with the goal of abutting, but not penetrating the cortex on the opposite side. A 0.7 mm diameter K-wire (IMEX Veterinary, Inc., Longview, TX) was placed in a pin vice and used to provide a pathway for a 0.6 mm diameter fiber optic temperature sensor (Neoptix, Quebec City, Canada), placed parallel to the axis of the spinal column, approximately 10 mm ventral from the dMWA applicator, and about in-line with the cross-section of maximum heating. A second fiber optic temperature sensor was placed in the spinal canal outside of the spinal cord dura mater, parallel to the first temperature sensor and in the same cross-sectional place as the dMWA applicator and was held in direct contact with the ventral wall of the canal by a cotton swab. Figure 2 shows the experimental setup for the *ex vivo* vertebrae study.

A peristaltic pump (Pump drive model #07528–30, pump head model #07514–10, Cole-Parmer, Vernon Hills, IL) circulated cooling water from an ice bath through the dMWA applicator at a flowrate of approximately 50 ml/ min. Ablations were performed at 120 W (generator setting) for 5 min (*n* = 6) and 80 W (generator setting) for 3.5 min (*n* = 8) for the room temperature samples and at 80 W (generator setting) for 3.5 min for the 37 °C samples (*n* = 6) (2450 MHz, 250 W, STARTER Evaluation kit, LEANFA Srl, Ruvo di Puglia, Italy). Ablations were conducted within 3–5 h after euthanasia without refrigeration or freezing of the samples.

Post-ablation, the vertebrae were cross-sectioned with a bandsaw along the transverse plane (parallel to the probe pathway), at or immediately adjacent to the dMWA applicator pathway. Measurements were taken of the grossly visible ablation zone, characterized by a white or gray discoloration of the marrow-filled cancellous bone tissue. Measurements included the dorsoventral distance perpendicular to the shaft (ablation depth) and laterolateral distance extending in parallel to the applicator shaft (ablation height). Two specimens were further sectioned along the sagittal plane to give a grossly visible measurement of the ablation width. Eleven of the specimens, including the two longitudinal sections, were fixed in 10% neutral buffered formalin, decalcified in 5% formic acid solution, embedded in paraffin, sectioned 5 µm thick, and hematoxylin and eosin (H&E) stained for histologic analysis by light microscopy. H&E stained slides were digitally scanned at 40X objective magnification and uploaded into Aperio eSlide Manager (Leica Biosystems, Buffalo Grove, IL) for imaging and measurements.

**Figure 3.**
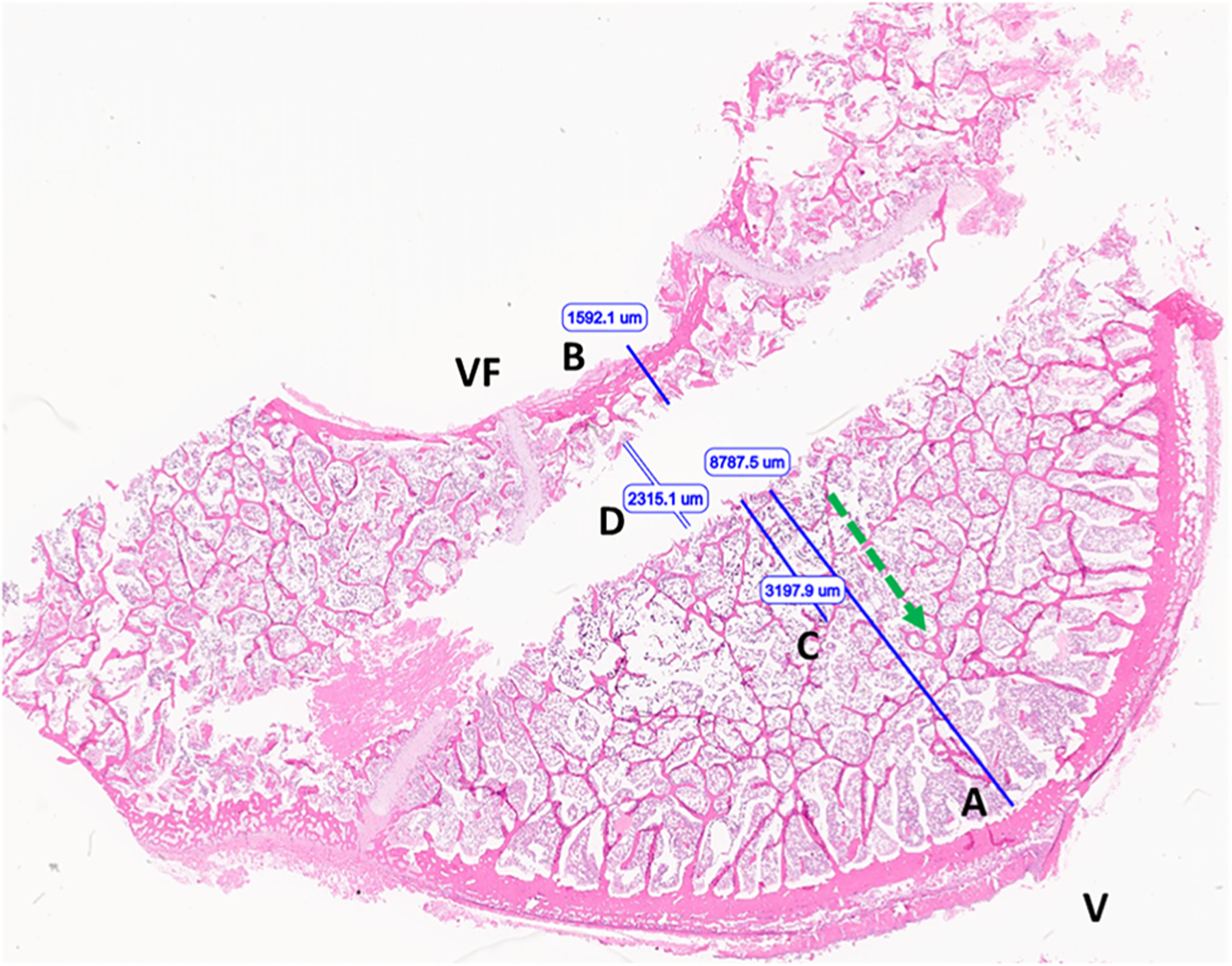
Image of vertebral body following *ex vivo* microwave ablation (H&E stained section). optimal T1 placement distance (A); optimal T2 placement distance (B); extent of nuclear streaming/precipitation (necrosis) (C); applicator tract width (D). Ventral surface (V), vertebral foramen/dorsal aspect (VF), ablation direction (green arrow).

The specific locations of the T1 and T2 temperature sensors were not identifiable on histologic evaluation. The eSlide Manager caliper tool was used to measure the perpendicular distance from the margin of applicator tract to the endosteal surface of the cortex in the forward direction (optimal T1 placement) and the perpendicular distance from the applicator tract to the periosteal surface of the spinal canal (optimal T2 placement), as shown in Figure 3. These measurements were taken to set the absolute maximum distance T1 could have been positioned and the absolute minimum distance T2 could have been positioned for comparison to the distances recorded during the *ex vivo* experiments and ultimately the distances used to select final T1 and T2 temperatures from the computational models.

### II.4 Pilot ablation studies in porcine vertebral body in vivo

The *in vivo* pilot experiment was carried out at the KSU College of Veterinary Medicine under an experimental protocol approved by the KSU Institutional Animal Care and Use Committee. The experiment was conducted in an approximately 45 kg, female domestic swine. Anesthesia was induced by an intramuscular injection of 4.4 mg/kg Telazol, and 2.2 mg/kg Xylazine. The pig was orotracheally intubated and anesthesia was maintained by isofluorane in O_2_.

#### II.4.1 CT imaging technique

The pig was placed in sternal recumbency to facilitate a dorsal approach for the ablation procedure. A lumbar survey CT scan was completed for baseline images of the lumbar vertebral bodies and musculature prior to intervention. Individual lumbar vertebral bodies were selected for introduction of the dMWA applicator and the temperature sensor. Short scans 7.5–11 cm in length centered at the level of the selected vertebra were repeated with each advancement or re-orientation of instrumentation to guide placement at the correct angle and depth within the vertebral body.

Data were acquired using a 16-row multidetector CT scanner (Bright Speed, General Electric Healthcare, Chicago, IL) in a helical manner with the following parameters: 120 kVp, 19–156 mAs, pitch factor 0.5625, 512 × 512 matrix, gantry rotation speed 0.6 s, slice thickness 0.625–5 mm. Images were acquired in soft tissue and bone windows and reconstructed in dorsal and sagittal planes.

#### II.4.2 Surgical technique

The vertebral bodies to be ablated (*n* = 4; L1, L3, L4, L5) were identified by CT scan. The goal was to insert the dMWA applicator into the center of the vertebral body through the vertebral pedicle, cranial to the transverse process. This site was chosen from the cross-section CT slices and the corresponding site on the pig was determined using the axial CT aiming laser. A #11 scalpel blade was used to incise the skin 4–8 cm from dorsal midline. The distance from midline varied depending on the cross-sectional shape of the vertebral body selected and the insertion angle required to place the antenna into the center of the vertebral body. A 10 cm, 11 ga Jamshidi needle (CareFusion, Vernon Hills, IL) was inserted through the stab incision and epaxial musculature using a cross sectional CT image as a guide. A repeat CT scan was acquired upon contact of the Jamshidi needle with the vertebral pedicle to confirm correct placement and direction of insertion. The needle was advanced through the vertebral pedicle into the vertebral body using serial CT scans to confirm the correct placement of the needle. After the CT scan confirmed the insertion of the Jamshidi needle to the depth of the ventral cortex of the vertebral body, the needle was withdrawn to the level of the transverse process, the trocar point stylet was removed, and the microwave antenna inserted. The placement process was repeated on the contralateral side for insertion of a 4-point fiber optic temperature sensor (FISO Technologies, Quebec, Canada).

150 W of microwave (MW) power, as read from the MW module in the generator, was applied for 5 min to L3, L4, and L5. 150 W of MW power was applied for 7.5 min to L1 after technical issues prevented any indications of ablative temperatures being reached in L3 and L4 and the temperature readings in L5 suggested adding duration to the procedure was possible. L2 was left unablated and designated as a control specimen.

#### II.4.3 Assessment of ablation zones by MRI imaging and histopathology

The pig was euthanized at the conclusion of the ablation procedure while still under general anesthesia. Euthanasia was performed by intravenous injection of sodium pentobarbital 390 mg/ml at a rate of 1 ml/5 kg of body weight. Following euthanasia, the lumbar spinal column and paraspinal musculature (epaxial and hypaxial) were removed en bloc post procedure and placed on top of a 16 channel Flex Speeder coil (Canon Medical Systems, Tustin, California) with the ventral aspect along the coil. This was isocentered within a 3 T magnetic resonance scanner (Galan 3 T, Canon Medical Systems, Tustin, California). T1-weighted, T2-weighted and T2-weighted with fat saturation sequences were performed in axial, sagittal and dorsal (T1-weighted only) planes. Acquisition parameters were as follows: T1-weighted (TR: 731 or 750 ms, TE: 10 ms, number of averages: 3, 2.5 mm slice thickness, 2.7 mm interslice gap), T2-weighted (TR: 14780 ms, TE: 80 ms, number of averages: 2, 2.5 mm slice thickness, 2.7 mm interslice gap), T2-weighted with fat saturation (TR: 15294 ms, TE: 80 ms, number of averages: 3, 2.5 mm slice thickness, 2.7 mm interslice gap). Data were stored in digital imaging and communications in medicine (DICOM) format on a viewer and patient archive system (Carestream, Rochester, New York).

Following MRI imaging, the vertebrae were cross-sectioned with a bandsaw along the axial plane at or immediately adjacent to dMWA applicator pathway. The gross specimens were visually inspected and photographed. The specimens were then fixed in 10% neutral buffered formalin, decalcified in 5% formic acid solution, embedded in paraffin, sectioned 5 µm thick, and hematoxylin and eosin (H&E) stained for histologic analysis by light microscopy.

## III. Results

**Figure 4.**
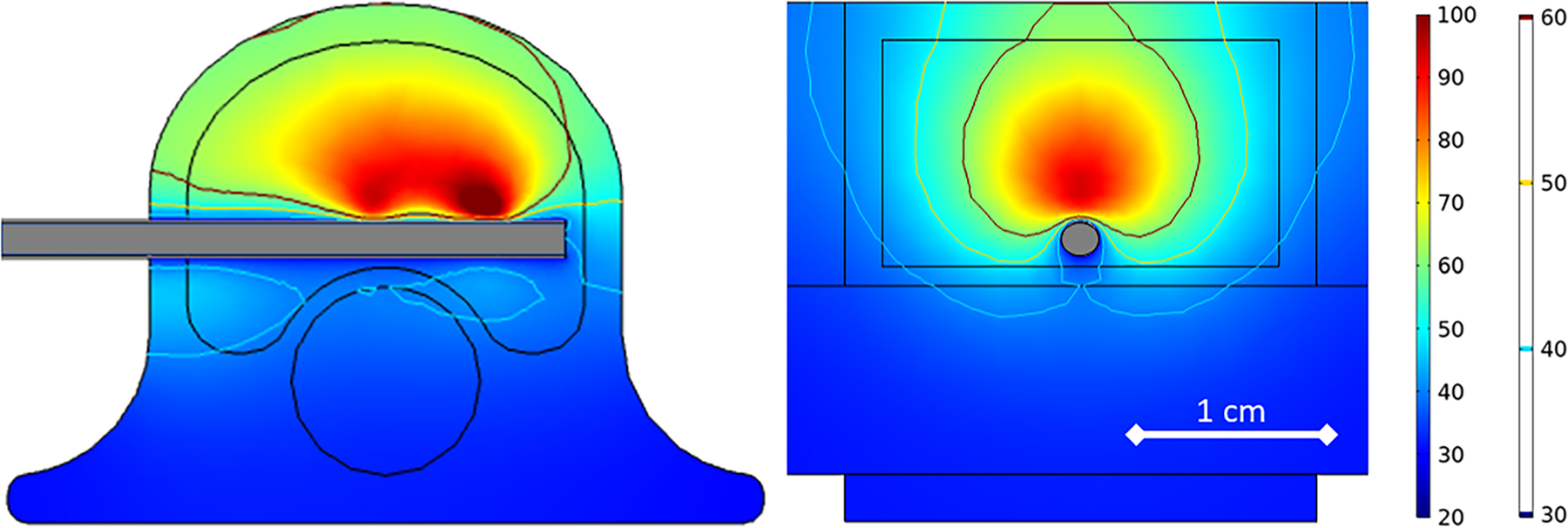
80 W, 3.5 min *ex vivo* ablation simulation with initial tissue temperature of 20 °C.

The simulated temperature at a location of 9.5 mm perpendicular from the dMWA applicator in the forward direction (T1), and 2.5 mm from the dMWA applicator in the backward direction (T2) for the *ex vivo* computational models are listed in Table 2. The extents of the simulated 55 or 60 °C isothermal contour did not correlate well to the region of visible discoloration in the experimental results, as occurs in soft tissue, such as liver. Simulated final temperature distribution of the 80 W, 3.5 min, 20 °C initial temperature model is shown in Figure 4.

**Figure 5.**
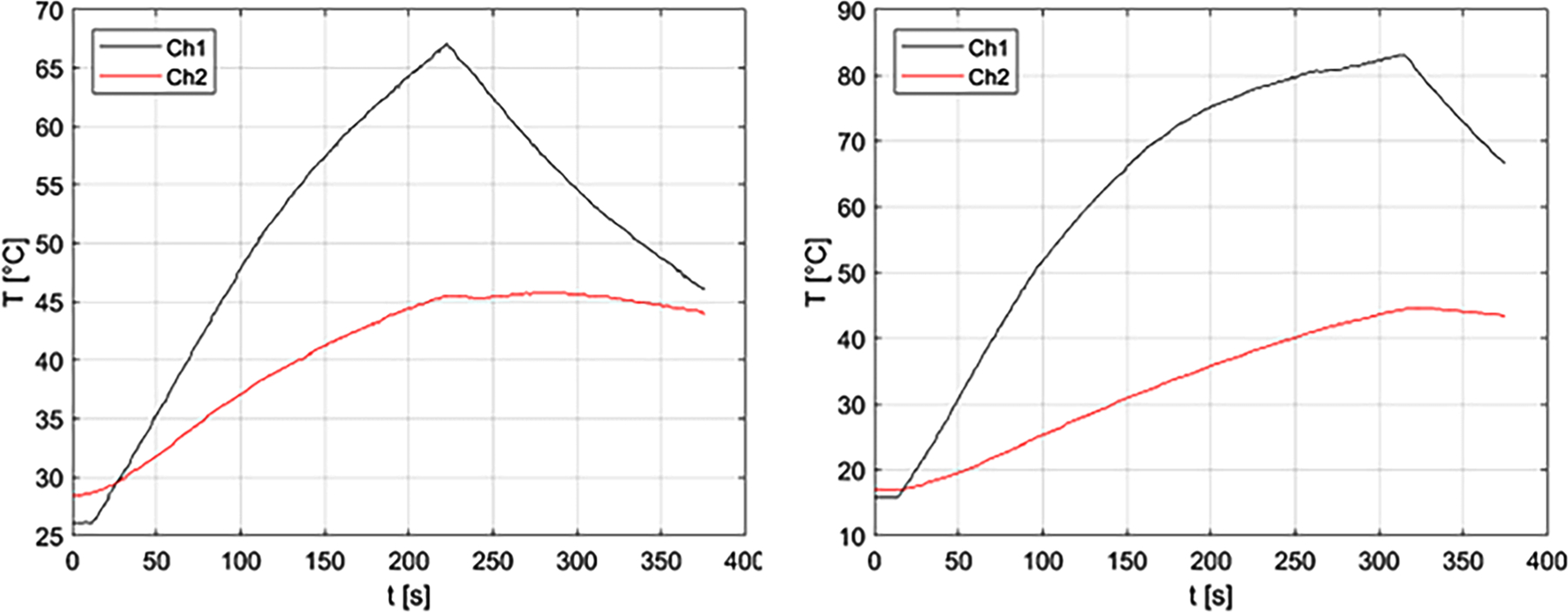
Experimentally measured T1 (Ch1) and T2 (Ch2) during 80 W, 3.5 min ablation (left) and during 120 W, 5 min ablation (right) in *ex vivo* porcine vertebral body.

For the *ex vivo* experiments, measurements of the grossly visible ablation zone, as defined by the whitened or grayed region, are provided in Table 3 as D (radial depth) and L (longitudinal length). Initial, final, and ΔT1 and ΔT2 measurements, as well as sensor placement distance (measured from the dMWA applicator) are also provided in Table 3. One set of T1 and T2 measurements was excluded from the 120 W experimental set due to a data logging error. Plots showing the ΔT1 and ΔT2 measurements during an 80 W, 3.5 min ablation and a 120 W, 5 min ablation are provided in Figure 5. Reflected power was less than 10% in all cases.

The T1 distance averaged among all experimental measurements was 9.5 mm. The maximum possible T1 distance measured and averaged among all histology slides was 10.1 mm with a range of 8.3 to 12.7 mm. The T2 distance averaged among all experimental measurements was 4.8 mm. The minimum possible T2 distance measured and averaged among all histology slides was 2.4 mm with a range of 1.4 to 3.6 mm.

The comparison of simulated vs experimentally measured T1 and T2 temperatures are provided in Table 4.

**Figure 6.**
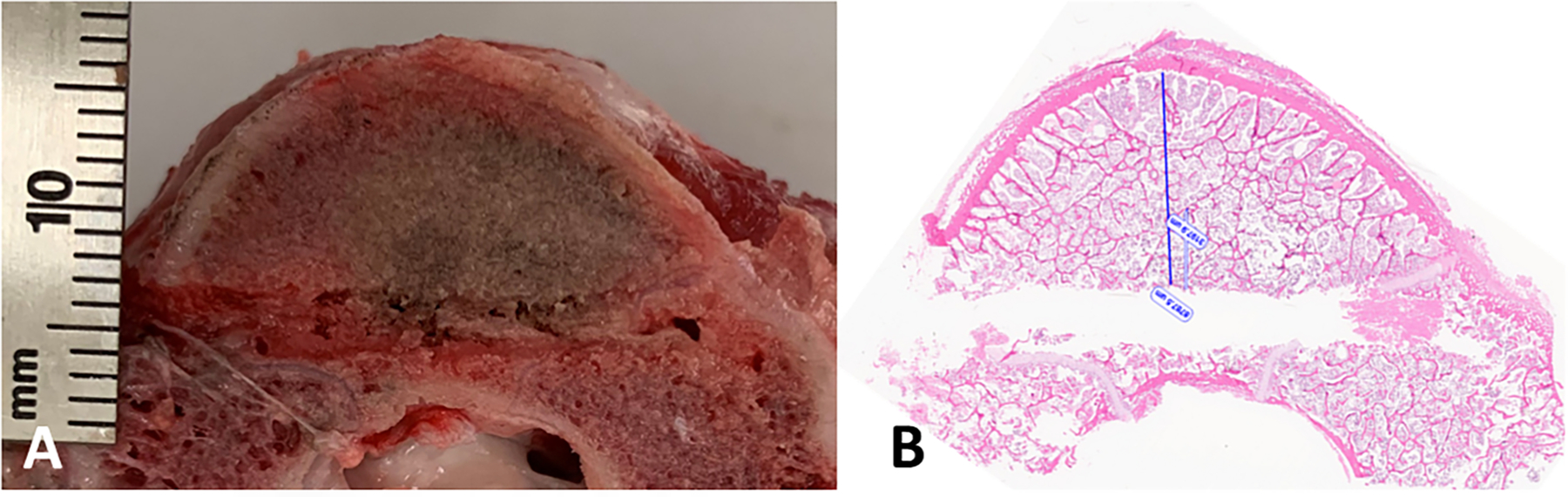
Transverse cut of vertebra, along the DMWA applicator pathway. Ablation conditions: 120 W, 5 min, room temperature initial temperature *ex vivo* ablation zone (A) Corresponding H&E stained section (B).

**Figure 7.**
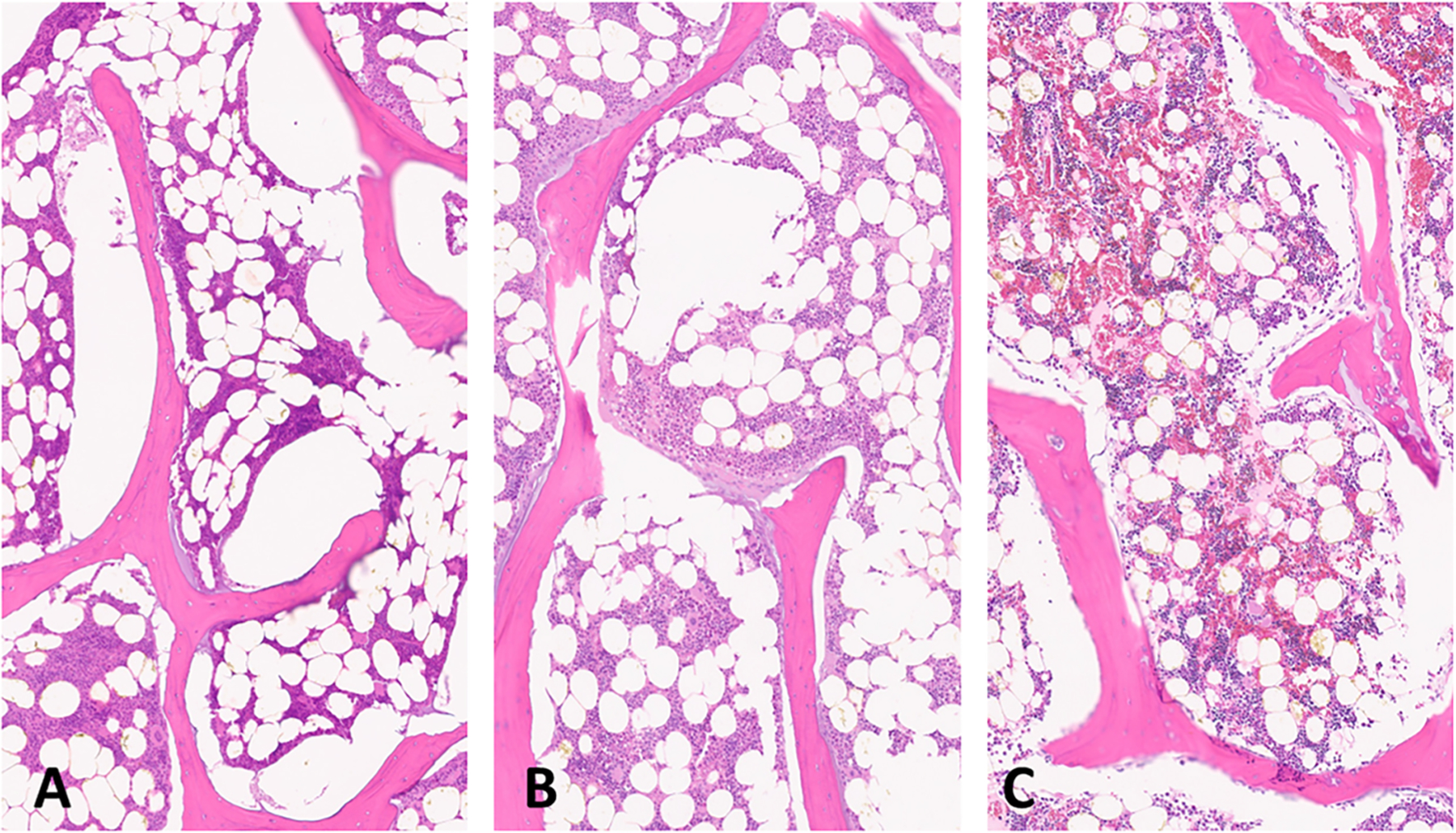
Sections of ablated and unablated vertebrate. Area immediately adjacent to DMWA applicator antenna heating showing severe bone marrow coagulation necrosis characterized by complete loss of cellular detail, as well as precipitation and clumping of cell remnants, coagulation of serum, erythrocytes, and marrow stroma (A). Area adjacent to (A) exhibiting less severe coagulative necrosis characterized by diffuse pallor and only partial loss of cellular details (individual cells and their nuclear detail are retained), as well as mild precipitation and clumping of cell remnants, coagulation of serum, erythrocytes and marrow stroma (B). Normal vertebrae from an unablated specimen having preservation of myeloid and lymphoid precursors and erythrocytes, and retention of histologically normal serum (C). (5 µm, H&E stained sections, 100× magnification).

A longitudinal section of an experimental *ex vivo* ablation zone is shown grossly (A) and histologically (B) in Figure 6. Representative images of H&E stained vertebral bone sections at 100x magnification demonstrate histologic changes of intensely heated (A), moderately heated (B), and unheated (C) bone tissue in Figure 7. Near the dMWA applicator (Figure 7A), intense heating caused severe coagulative to lytic -type necrosis with hypereosinophilia and loss of cellular detail including hematopoietic and adipose tissue, erythrocytes, and precipitation and clumping of cellular debris. In regions more distant from the dMWA applicator (Figure 7B), moderate heating caused less severe coagulative-type necrosis characterized by erythrocyte and hematopoietic cell lysis and hypereosinophilia and pallor (as seen in intensely heated regions), though with overall preservation of hematopoietic and erythroid precursors, and some erythrocytes. Also shown is an unablated marrow space from a normal vertebral body, where there is no evidence of necrosis, cellular detail of mature erythrocytes and hematopoietic precursors are well preserved and recognizable (Figure 7C).

For the *in vivo* pilot study, it was determined the dMWA applicator was not inserted far enough during the first two ablations (L3 & L4) and therefore the vertebral body was shielded from the MW energy by the metal shaft of the Jamshidi introducer. During the second two ablations (L1 & L5) a temperature rise was recorded by the fiber optic sensor indicating ablative temperatures were reached in portions of the vertebral body but were not logged due to inadvertent operator error. Gross visual inspection of the L1& L5 section did indicate some gross changes including mild pallor in the region of expected thermal damage, but the coloration change was too indiscrete to accurately measure.

**Figure 8.**
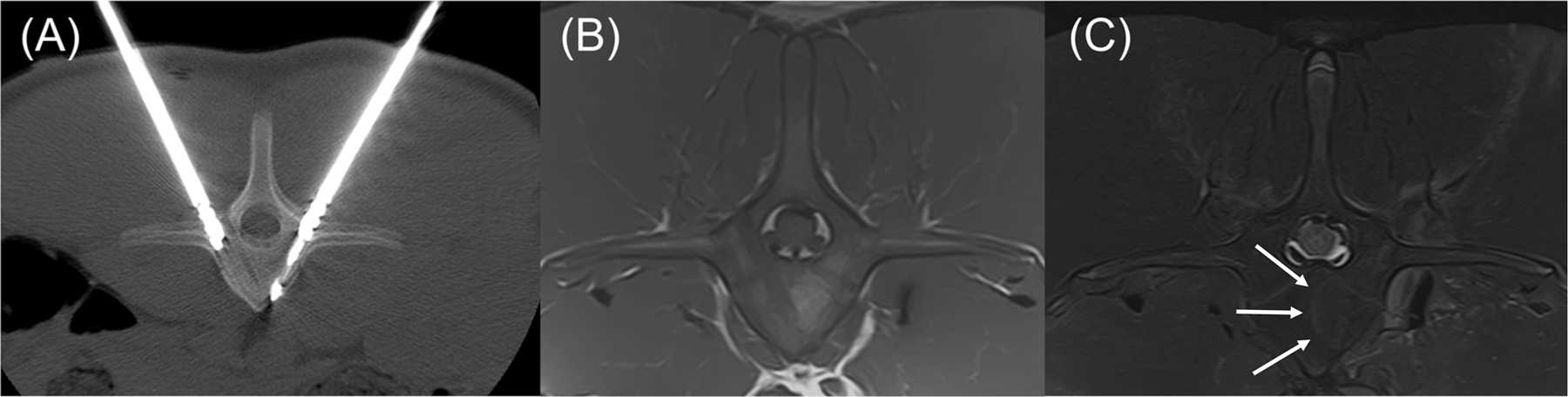
Transverse CT and MRI images of the L1 vertebra with anatomic right on the left side of the images. (A) CT image with dMWA applicator in the left aspect of the vertebral body and fiberoptic sensor in the right. (B) T1-weighted MRI image of L1 showing focal, semi-circular region of hyperintensity axial to the tract previously occupied by the dMWA applicator. (C) T2-weighted MRI image with fat saturation. Note the faint hyperintense rim along the peripheral aspect of the hyperintense region seen in the T1-weighted image (outlined by white arrows).

In T1-weighted, axial images of the cranial to mid aspects of the L1 vertebra there are 3 approximately 3.5–4.0 mm hypointense linear tracts extending from the pedicles to the ventroaxial aspect of the vertebral body (two right-sided, one left-sided), which are consistent with ablation applicator and sensor insertion sites. Within the left aspect of the vertebral body, just axial to the left-sided tract, is an approximately 16 mm long by 7 mm thick, semicircular area of focal, well defined T1-weighted hyperintensity corresponding to the ablation zone. Within the same region of the L2 vertebral body, where there was no intervention, there is a more homogeneous, intermediate intensity that is slightly hyperintense to surrounding musculature. In T2-weighted sequences with fat saturation, there is a thin, hyperintense line along the convex, peripheral margin of the previously described ablation zone within the central portion of the vertebral body (Figure 8). Similar changes were noted in the L5 vertebral body.

**Figure 9.**
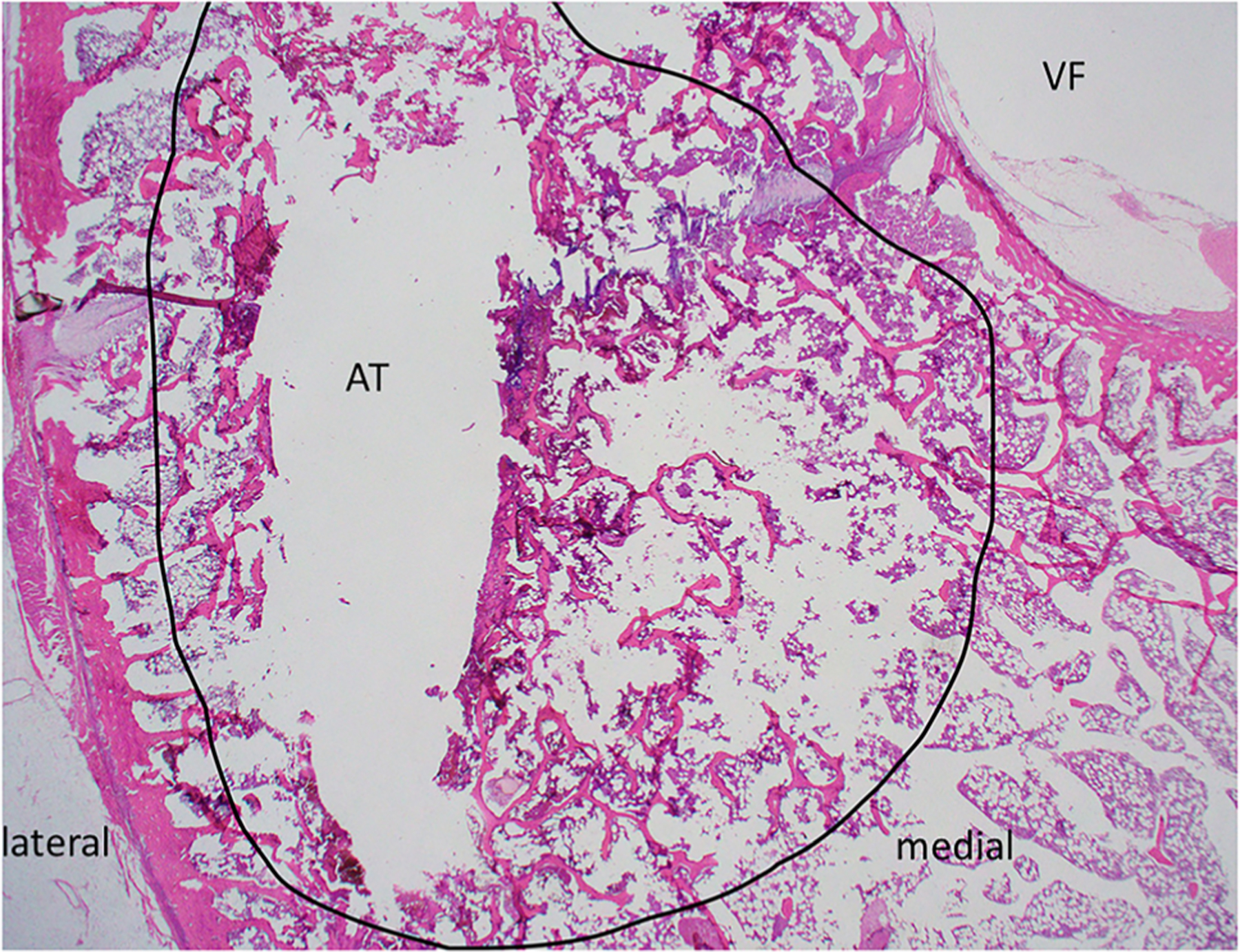
H&E stained slide of transverse section of ablated vertebrae L5. Medial and lateral margins are shown for orientation; the vertebral foramen (VF) and ablated tract (AT) are indicated. The region of thermal damage is circled in black.

Histologic sections of L1 were too fragmented for analysis. In the tangential histologic section of the L5 vertebral body, two linear tracts created by the Jamshidi needles to introduce the temperature sensor and dMWA applicator extended from the cortex of the periosteal surface of the dorsal lamina on both left and right sides of the dorsal spinous process, and extended ventro-medially through the left and right pedicles adjacent to but not entering into the vertebral foramen (Figure 9, VF). The mechanical injury associated with insertion of the temperature sensor (unablated tract) was characterized by a thin layer of coagulative necrosis affecting bony trabecula, bone marrow, and growth plate cartilage along and parallel to both the medial and lateral margins of the insertion tract. This area measured approximately 500– 600 µm thick on both medial and lateral margins of the tract, and was characterized by hypereosinophilia and coagulation of cells, with loss of cellular nuclear detail, and accumulation of homogenous eosinophilic fluid (not shown). In contrast to the unablated tract, the shape of the tissue injury along the ablation probe was distinct, especially within the vertebral body along the medial side of the ablated tract (Figure 9, AT). The affected region (Figure 9, area depicted by black circle) was comprised of a poorly demarcated unencapsulated oval to oblong region that measured up to 3.5 mm at the widest point from the linear tract medially toward the vertebral foramen (Figure 9, VF) and ventrally to the vertebral body; affected tissues along the ablated area include trabecular bone, bone marrow elements including hematopoietic cells and adipocytes, and cartilage of the growth plate. Distinct changes correlating with the altered appearance of tissues observed in T1 or T2-weighted scans was not evident in these sections by histopathologic examination; this is likely due to the insensitivity of light microscopy to detect subtle changes in fluid or tissue architecture which may be further altered or eliminated during routine tissue processing for histologic evaluation (fixation, dehdyration, paraffin embedding, etc.).

## IV. Discussion

The interactions of microwaves in soft tissues (liver, lung, kidney, etc.) throughout the course of an MWA procedure, including how the biophysical properties of those tissues change at elevated temperatures, has been studied extensively and reported in the literature. However, how microwaves interact in bone and how bone tissue biophysical properties change during thermal ablation has not been as extensively presented in the literature. This experimental and computational modeling study was initiated to study thermal profiles in bone during microwave ablation.

First, there was a large difference in the extents of the simulated 55 or 60 °C isothermal contours compared to the extents of the visibly discolored regions in the *ex vivo* experimental tissue specimens post-ablation, with the visibly discolored regions being much smaller than the simulated 55 or 60 °C contours. The *ex vivo* experiments, which confirm average T1 temperatures rose to 53.4 − 80.3 °C approximately 9.5 mm in the forward-firing direction from the applicator, suggest the grossly visible region of discoloration in *ex vivo* bone is not as good an indicator of the extents of the 55–60 °C heating contour as it is in other soft tissues. This may be due to reduced or different mechanisms of changes in the physical properties of the bone tissue that otherwise lead to coloration changes in soft tissues at ablative temperatures. The discoloration or pallor of the ablation zone observed grossly may be caused by lysis of red blood cells, which occurs throughout a region extending histologically slightly beyond the macroscopically visible discolored region. Further confounding these results, the boundary of visible discoloration in the bone ablation samples is quite faint, making it difficult to accurately measure, and the process of using a bandsaw (with associated tissue distortion and heating) to section the *ex vivo* vertebrae may alter the cut surface of the specimen, impacting accurate gross and histologic assessment of ablation zone extents.

Given that bone tissues already have a relative permittivity and electrical conductivity about one half (cancellous bone) to one quarter (cortical bone) of most soft tissue [15], this study made an initial assumption that those values would not change appreciably during thermal ablation (bone has less water content to dehydrate, fewer proteins to desiccate, and 70% inorganic hydroxyapatite constitution). As previously discussed, because the extent of the grossly visible ablation zone cannot be reliably used as an indicator of ablation zone temperature without further study, the temperature data recorded from experiments were used to evaluate the validity of the computational models.

However, since each vertebra has a non-uniform size and shape and placement of the dMWA applicator and the temperature sensors had to be referenced externally to the vertebrae at the time of the experiments, there was significant uncertainty as to the exact placement of the temperature sensors. Exact temperature probe placement would have a high impact on correlating simulated and experimental results. The exact placement of T1 and T2 during the ablation experiments could not be identified histologically, though the maximum possible T1 and minimum possible T2 placement distances could be measured. Based on histologic measurement, the maximum T1 distance had a mean of 10.1 mm, which means the mean distance of 9.5 mm for T1 placement recorded during the ablation procedure was reasonable. A much larger discrepancy was observed between the average T2 distance measured during the ablation procedure (4.8 mm) and the optimal minimum T2 distance measured from the histology slides (2.4 mm). This discrepancy may partially be a result of the spinal canal having a non-uniform diameter though a vertebra (it widens toward the center), which is not accounted for with externally referenced distance measurements. The optimal minimum T2 distance measured and averaged from the histology slides was likely more representative of actual T2 placement than what was measured during the experiments and therefore was used to determine the simulated T2 temperatures. For both T1 and T2, there could also have been errors in placing the temperature sensors exactly within the same vertebral cross-sectional plane as the dMWA applicator. An analysis of this range of possible error could not be reconstructed post-experiment, but in general, less than optimal placement would have resulted in lower experimental T1 temperatures, but potentially higher experimental T2 measurements due to the sidelobes of the dMWA applicator heating pattern [12].

Though it is possible bone tissue properties exhibit some temperature dependent changes during microwave ablation similar to other tissue types, the relatively minor differences in simulated vs. experimental temperatures shown in this study (at T1 and T2, Table 4) suggest a static bone tissue property model could be a reasonable starting point for developing an *in vivo* model of using dMWA to treat metastatic disease in human vertebrae. However, additional experimental work to measure the temperature dependent dielectric and thermal properties of bone is needed.

It is important to note that the pigs used in the experimental study were young and their vertebral bodies have a slightly different tissue and mineral composition than that of a more mature animal [17]. Further study should be conducted to determine if a different tissue composition within the vertebral body would have a meaningful impact on the microwave ablation zone when treating metastatic disease in elderly patients.

Although T1 temperatures in the range (~90–100 °C) observed in *ex vivo* simulations and experimentation may be concerning when considering the need for spatial control of thermal damage in clinical use, these high T1 temperatures may be impacted by the experimental setup where a relatively small tissue sample was surrounded by air. Air is a thermally insulative material compared to soft tissues, which means much of the heat input within the tissue specimen would be contained in it causing elevated temperatures. In our prior preliminary studies, simulations in which the surrounding medium of air was replaced with muscle showed a much lower final temperatures at T1 (36.4–52.6 °C for the MW power and duration combinations presented in Table 2) [11]. Further *ex vivo* experimentation could be conducted with more soft tissues surrounding the vertebral specimens, however, the surrounding soft tissue will make exact placement and measurement of the dMWA applicator and temperature sensors even more challenging.

## V. Conclusion

Over the range of ablation power/duration combinations examined, there was a 3–7% difference in experimental vs. simulated changes in T1 temperatures (Table 4). The difference in experimental vs. simulated changes in T2 temperatures was higher at 11–37% (Table 4). Given the relatively minimal differences observed between the simulated and experimental data, a static tissue property model could be instructive for simulating MWA in bone. The temperature dependence of bone tissue is currently unknown, but could be a source of future work to further improve the model. Pilot *in vivo* studies in porcine vertebral bodies show dMWA has potential for clinical application treating metastatic tumors in the vertebral body and potentially other bone sites.

## Figures and Tables

**Table 1.** Tissue biophysical properties employed for modeling 2.45 GHz microwave ablation in *ex vivo* porcine vertebral body [15].

Parameter	units	cortical bone	cancellous bone	cartilage	muscle	spinal cord
Relative permittivity		11.4	18.5	38.8	52.7	30.1
Electric conductivity	S/m	0.39	0.81	1.76	1.74	1.09
Thermal conductivity	W/m/C	0.32	0.31	0.49	0.49	0.51
Density	kg/m^3^	1908	1178	1100	1090	1075
Specific heat capacity	J/kg/C	1313	2274	3568	3421	3630

**Table 2.** Simulated *ex vivo* bone ablation results.

P (W)	Time (min)	T_i_ (°C)	T1_f_ (°C)	ΔT1 (°C)	T2_f_ (°C)	ΔT2 (°C)
80	3.5	20	51.3	31.3	33.4	13.4
80	3.5	30	62.5	32.5	39.6	9.6
80	3.5	37	68.9	31.9	43.8	6.8
120	5	20	81.4	61.4	41.8	21.8

Ti is the pre-ablation temperature of the bone tissue, T1_f_ is the temperature at sensor T1 at the end of the ablation, ΔT1 is the difference between T_i_ and T1_f_, T2_f_ is the temperature at sensor T2 at the end of the ablation, ΔT2 is the difference between T_i_ and T2_f_.

**Table 3.** *Ex vivo* vertebrae ablation experimental results (mean ± std).

			Visible ablation								Temp sensor placement	
P (W)	t (min)	*n*	D (mm)	L (mm)	T1_i_ (°C)	T1_f_ (°C)	ΔT1 (°C)	T2_i_ (°C)	T2_f_ (°C)	ΔT2 (°C)	D_T1_ (mm)	D_T2_ (mm)
80	3.5	7	3.0 ± 0.5	9.0 ± 1.5	19.9 ± 0.8	53.4 ± 2.9	33.5 ± 2.9	20.9 ± 0.5	35.9 ± 1.5	15.0 ± 1.5	9.3 ± 0.9	4.3 ± 0.0
80	3.5	6	5.5 ± 0.5	10.5 ± 0.8	30.2 ± 3.7	63.7 ± 9.8	33.5 ± 9.1	35.1 ± 4.0	45.9 ± 4.3	10.8 ± 5.8	9.3 ± 1.0	5.3 ± 0.5
120	5	6*	7.8 ± 1.0	14.0 ± 2.0	17.2 ± 1.7	80.3 ± 9.8	63.1 ± 9.5	17.5 ± 1.2	49.8 ± 4.8	32.3 ± 4.1	9.8 ± 1.0	5.0 ± 0.0

T_i_ is the pre-ablation temperature of the bone tissue, T1_f_ is the temperature at sensor T1 at the end of the ablation, ΔT1 is the difference between T_i_ and T1_f_, T2_f_ is the temperature at sensor T2 at the end of the ablation, ΔT2 is the difference between T_i_ and T2_f_.

*one sample was excluded from the T1 and T2 measurements.

**Table 4.** *Ex vivo* vertebra ablation experimental vs. simulated ΔT1 and ΔT2.

Power (W)	Time (min)	T_bone_ (ideal) (°C)	Experiment ΔT1(°C)	Simulation ΔT1 (°C)	Δexp-sim	Experiment ΔT2 (°C)	Simulation ΔT2 (°C)	Δexp-sim
80	3.5	20	33.6	31.3	−7%	15	13.4	−11%
80	3.5	37	33.5	31.9	−5%	10.8	6.8	−37%
120	5	20	63.2	61.4	−3%	32.3	21.8	−33%

## Data Availability

The data that support the findings of this study are available from the corresponding author upon reasonable request.
